# *KRAS*^G12C^突变非小细胞肺癌诊断及靶向治疗进展

**DOI:** 10.3779/j.issn.1009-3419.2025.101.13

**Published:** 2025-08-20

**Authors:** Jiahe SHI, Yufang WANG, Jing ZHENG, Jianya ZHOU

**Affiliations:** 310000 杭州，浙江大学医学院附属第一医院呼吸与危重症医学科; Department of Respiratory Medicine, The First Affiliated Hospital, College of Medicine, Zhejiang University, Hangzhou 310000, China

**Keywords:** Kirsten鼠类肉瘤病毒癌基因, 肺肿瘤, 基因检测, 靶向治疗, Kirsten rat sarcoma viral oncogene, Lung neoplasms, Genetic testing, Targeted therapy

## Abstract

肺癌是全球范围内癌症相关死亡的首要原因，对人类健康构成重大威胁。非小细胞肺癌（non-small cell lung cancer, NSCLC）患者中，Kirsten鼠类肉瘤病毒癌基因（Kirsten rat sarcoma viral oncogene, *KRAS*）突变是重要的致癌驱动因素，其中*KRAS*^G12C^突变是最常见的亚型之一。目前，*KRAS*突变的检测方法主要集中在聚合酶链式反应（polymerase chain reaction, PCR）及测序平台，两类平台所衍生出的多种技术在检测性能和检测通量等方面各有优劣，并在组织活检及液体活检领域具有重要应用价值。在靶向治疗方面，索托拉西布、阿达格拉西布、氟泽雷塞、格索雷塞、戈来雷塞等*KRAS*^G12C^靶向药物在临床试验中展现出一定疗效，先后获批应用于临床。为克服靶向药物的耐药性，改善患者获益，靶向药物与化疗、免疫检查点抑制剂（immune checkpoint inhibitors, ICIs）、Src同源2结构域蛋白酪氨酸磷酸酶2（Src homology region 2 domain-containing phosphatase 2, SHP2）抑制剂、表皮生长因子受体（epidermal growth factor receptor, EGFR）单克隆抗体等药物的联合治疗策略不断涌现。本文系统回顾了*KRAS*^G12C^突变NSCLC的诊断及靶向治疗进展，旨在为临床治疗策略的选择及后续研究提供参考。

## Abstract (en)

Lung cancer stands as the primary cause of cancer-related mortalities globally, presenting a severe menace to human health. In individuals with non-small cell lung cancer (NSCLC), Kirsten rat sarcoma viral oncogene (*KRAS*) mutations serve as crucial oncogenic drivers. NSCLC with *KRAS*^G12C^ mutation is among the most prevalent subtypes. Currently, the detection methods for *KRAS* mutations predominantly concentrate on polymerase chain reaction (PCR) and sequencing platforms. The diverse derivative technologies of these two platforms each exhibit distinct merits and demerits in terms of testing performance and detection throughput, and find significant applications in tissue biopsy and liquid biopsy. In targeted therapies, *KRAS*^G12C^ targeted drugs, including Sotorasib, Adagrasib, Fulzerasib, Garsorasib, and Glecirasib, have demonstrated certain therapeutic efficacies in clinical trials and have obtained marketing approval. To tackle drug resistance and enhance patient’s prognoses, combination therapeutic strategies that integrate targeted agents with chemotherapy, immune checkpoint inhibitors, Src homology region 2 domain-containing phosphatase 2 (SHP2) inhibitors, and epidermal growth factor receptor (EGFR) monoclonal antibodies have emerged. This paper systematically reviews the advancements in the diagnosis and targeted therapy of NSCLC with *KRAS*^G12C^ mutation, aiming to offer a reference for the selection of clinical treatment regimens and subsequent research.

肺癌是全球发病率和死亡率最高的恶性肿瘤之一。国际癌症研究数据^[1]^表明，全球每年新发肺癌病例248.03万，死亡人数181.72万。2022年，肺癌在我国的发病率和死亡率分别为75.13/10万和51.94/10万，已成为我国男女发病率及死亡率均最高的癌症^[2]^。非小细胞肺癌（non-small cell lung cancer, NSCLC）占全部肺癌的85%左右，其中腺癌是最常见的组织学亚型，约占40%^[1]^。作为驱动基因，Kirsten鼠类肉瘤病毒癌基因（Kirsten rat sarcoma viral oncogene, *KRAS*）突变在不同国家和地区之间存在显著差异。美国、欧洲等地肺腺癌*KRAS*突变发生率超过30%^[3]^，而我国肺腺癌*KRAS*突变发生率仅约12.8%^[4]^。G12C在各亚型*KRAS*突变中最为常见，于西方国家和我国分别占比约40%^[3]^和30%^[4]^。目前，我国*KRAS*突变NSCLC的一线治疗方案参考驱动基因阴性NSCLC方案，包括免疫检查点抑制剂（immune checkpoint inhibitors, ICIs）±化疗、化疗±抗血管生成治疗等^[5]^。临床研究^[6]^表明ICIs联合化疗治疗驱动基因阴性NSCLC患者中位无进展生存期（median progression-free survival, mPFS）为9个月，中位总生存期（median overall survival, mOS）为19.4个月。我国的回顾性研究^[7]^数据表明*KRAS*突变NSCLC患者的mOS可达22.9个月，其中基于ICIs的疗法疗效更佳。尽管如此，相较于部分靶向治疗已较为成熟的突变基因，针对*KRAS*突变的靶向药物研发曾长期陷入停滞状态——核心原因在于突变蛋白的结构特殊，无传统小分子药物结合的“成药口袋”。这一困境使得该部分患者的治疗之路充满挑战，远期预后始终不容乐观。近年来，针对*KRAS*^G12C^突变NSCLC的靶向药物研发取得重要突破，国产创新药也陆续获批上市，获得国内权威指南后线治疗的推荐，为患者带来新的希望^[5]^。本文旨在对*KRAS*^G12C^突变NSCLC近年来的诊断及靶向治疗进展进行综述，以期为临床用药及后续研究提供参考。

## 1 KRAS基因与NSCLC

### 1.1 RAS信号通路与KRAS基因

鼠类肉瘤病毒癌基因同源物（rat sarcoma viral oncogene homolog, RAS）家族是最早被发现的人类原癌基因之一，包括HRAS、NRAS和KRAS三种成员。其中KRAS是肺腺癌中常见的驱动基因之一，也是NSCLC致病机制的核心靶点之一^[8]^。

KRAS基因位于染色体的12p12.1区域，编码两个高度相关的蛋白异构体KRAS-4A和KRAS-4B^[8]^。KRAS蛋白是一种三磷酸鸟苷（guanosine triphosphate, GTP）酶，发挥信号通路传导开关的作用。其受到上游信号网络精密复杂的调控，主要包括生长因子、细胞因子和细胞外基质黏附信号等，通过激活相应的受体，如表皮生长因子受体（epidermal growth factor receptor, EGFR）/成纤维细胞生长因子受体（fibroblast growth factor receptor, FGFR）/间质上皮转化因子（mesenchymal-epithelial transition factor, MET）等受体酪氨酸激酶（receptor tyrosine kinases, RTKs）及整合素，招募并激活适配蛋白，如生长因子受体结合蛋白2、Src同源2结构域蛋白酪氨酸磷酸酶2（Src homology region 2 domain-containing phosphatase 2, SHP2），进而正调控鸟嘌呤核苷酸交换因子，或负调控GTP酶激活蛋白，从而对其开关功能实现精确控制^[8]^。而KRAS蛋白则通过交替结合GTP和二磷酸鸟苷（guanosine diphosphate, GDP），实现对丝裂原活化蛋白激酶（mitogen-activated protein kinase, MAPK）和磷脂酰肌醇3-激酶（phosphatidylinositol 3-kinase, PI3K）等关键下游信号通路的调控，从而调节细胞分化、增殖和代谢等过程^[8]^。

当KRAS基因突变后，其编码蛋白构象发生改变，GTP酶活性基本丧失，GTP从KRAS蛋白上解离减少，GDP与KRAS蛋白结合减弱，从而使KRAS蛋白一直处于活化状态，持续激活下游通路，造成细胞增殖失控、恶变，进而促进肿瘤的发生和发展^[9]^。

### 1.2 NSCLC中的*KRAS*突变

NSCLC中的KRAS基因突变类型多样，其中以密码子第12位的甘氨酸替换为半胱氨酸（G12C）、天冬氨酸（G12D）、缬氨酸（G12V）等点突变最为常见，其中G12C在各亚型中占比最高^[3]^。我国一项大样本研究^[4]^报告了*KRAS*突变NSCLC患者G12C、G12D和G12V的占比，分别为29.6%、18.1%和17.5%。*KRAS*^G12C^突变NSCLC在男性患者及吸烟患者中好发，具有较高肿瘤突变负荷，常伴随肿瘤抑制蛋白p53（tumor supressor p53, TP53）基因、EGFR基因、丝氨酸-苏氨酸激酶11（serine/threonine kinase 11, STK11）基因和Kelch样ECH关联蛋白1（kelch-like ECH-associated protein 1, KEAP1）基因共突变^[3,4]^。由此可见，*KRAS*^G12C^突变NSCLC明确的人群集中趋势、独特的分子背景等特点构成其在临床与科研中的独特地位，为精准治疗提供了关键切入点。

### 1.3 *KRAS*^G12C^成药机制的突破

KRAS蛋白其含有G结构域（催化结构域）和高变区两个区域，G结构域由开关I、开关II和P环三个区域组成，可结合GTP并激活信号传导，而高变区则驱动KRAS蛋白锚定到膜上^[10]^。在KRAS基因突变的个体中，由于KRAS蛋白与GTP能够以皮摩尔级别的亲和力相结合，小分子抑制剂难以竞争性抑制KRAS蛋白的活化，且KRAS蛋白本身结构决定其难以与小分子药物相结合，导致针对*KRAS*突变的靶向药物研发一度陷入停滞。然而，研究^[11]^发现*KRAS*^G12C^突变后，其蛋白G结构域开关II区形成了药物结合口袋（switch II pocket, S-IIP），为小分子靶向药物的研发提供了突破的可能。而其他常见突变亚型则因缺乏反应性半胱氨酸或空间构象不同，难以形成类似的共价结合口袋，但令人欣喜的是，已有多种针对其他亚型突变的KRAS靶向药物处于研发中（如KRAS^G12D^抑制剂、泛RAS抑制剂等），标志着*KRAS*突变靶向治疗已从“不可成药”的困境迈向了针对多种亚型的精准探索。

### 1.4 *KRAS*^G12C^靶向药物的耐药机制

在NSCLC中，目前已获批各类*KRAS*^G12C^靶向药物在临床研究中仅展示出对后线治疗患者28%-50%的客观缓解率（objective response rate, ORR），提示了显著比例的固有耐药，同时各药物的mPFS均不足1年，反映了获得性耐药同样普遍^[12-17]^。当前研究^[18]^表明，固有耐药与伴随基因改变密切相关，例如STK11和KEAP1共突变可能通过重塑肿瘤免疫微环境、激活抗氧化应激通路等方式降低对MAPK信号依赖。获得性耐药机制则更为多元：上游通路的激活，如RTKs（EGFR、MET等）的过表达或扩增，能通过SHP2及SOS1（son of sevenless homolog 1）等介导重新驱动RAS-MAPK信号级联；下游旁路的激活，如B-Raf原癌基因丝氨酸/苏氨酸蛋白激酶（B-raf proto-oncogene, serine/threonine kinase, BRAF）融合/扩增或裂原活化蛋白激酶激酶（mitogen-activated protein kinase kinase, MEK）突变，可绕过KRAS直接维持下游信号；RAS家族的其他突变，如*NRAS*^Q61K/L/R^和*HRAS*^G13V^可恢复被*KRAS*^G12C^抑制剂阻断的信号传递；KRAS基因扩增或二次突变（如R68S、H95D/G/N/R和Y96C/D/H/N等位点）可改变S-IIP的构型，从而降低抑制剂的结合力；组织学转化，如上皮-间质转化、腺癌向鳞癌/小细胞癌的转变等亦有可能参与耐药过程^[18]^。目前，已有多项联合疗法进行了克服耐药的尝试并取得一定积极结果，如与ICIs的联合以改善免疫微环境^[19]^、与EGFR单抗^[20]^/SHP2抑制剂^[21]^的联合以抑制旁路激活、与化疗联合以快速缩瘤并延缓耐药^[22]^等，具体将在本文“3.3 联合用药”进行阐述。

## 2 *KRAS*突变的诊断进展

目前，由于对诊断性能要求较高，针对NSCLC患者*KRAS*突变的检测主要集中在聚合酶链式反应（polymerase chain reaction, PCR）和测序平台，国内指南推荐PCR、二代测序技术（next-generation sequencing, NGS）作为KRAS基因的检测手段^[5]^。根据标本来源的不同，检测技术的选择也有所差异。

组织活检是肺癌确诊的“金标准”，而液体活检是通过分析体液中可能存在的肿瘤成分实现监测肿瘤基因状态的技术，其中循环肿瘤DNA（circulating tumor DNA, ctDNA）能准确反映KRAS基因的突变状态^[23]^。因ctDNA的浓度较低，液体活检需要通过高灵敏度的技术检测突变，但其相对无创、可重复检查的优势极大减轻了患者的负担，尤其适用于组织样本不可及、不足或质量差等场景，同时在肿瘤负荷评估、预后评估、早期筛查、术后监测等方向也有着广阔的应用前景^[24]^。精准的分子诊断有助于*KRAS*突变的分型，从而帮助患者选择更加合适的靶向药物，因此诊断技术的进步至关重要。

### 2.1 PCR及衍生方法

#### 2.1.1 PCR与实时荧光定量PCR（quantitative real-time PCR, qPCR）

PCR技术能实现特定DNA片段的指数级扩增，进而检测突变，但无法对目标片段进行定量分析。qPCR引入了荧光标记，能实时监测每轮扩增后产物的数量，实现了“相对定量”。新一代PCR方法不断优化，突破了传统技术在灵敏度和特异性的局限，提升了对低频突变的检测能力，并在定量能力上显著增强，从而更快速高效地完成样本分析。

#### 2.1.2 等位基因特异性PCR（allele-specific PCR, AS-PCR）/扩增阻滞突变系统-PCR（amplification refractory mutation system-PCR, ARMS-PCR）

AS-PCR根据等位基因特异性引物与靶序列的完全匹配，实现特异性延伸，结合qPCR等技术即可精确检测突变基因^[25]^。

AS-PCR在肺癌分子诊断中已形成成熟的应用体系^[26]^，多重AS-PCR能以较低成本和较少样本实现对NSCLC常见突变的快速、高灵敏、高特异性检测，性能优于Sanger测序及NGS，对KRAS的检测限可低于1%，但稀有突变的检测性能仍有待提升^[27-29]^。大样本荟萃分析^[30]^证实其作为液体活检手段的有效性，与组织活检相比，AS-PCR液体活检检测EGFR突变的敏感性、特异性、诊断比值比（diagnostic odds ratio, DOR）、受试者工作特征曲线下面积（area under the curve, AUC）分别为65.3%、98.2%、52.8、0.71，但在*KRAS*突变检测中的结论仍有待研究。

然而，该技术也存在局限性：需要针对新突变广泛优化反应条件和引物结构；无法检测未知突变；因反应进程的不确定性，需要在引物体系中加入非配对碱基以提升特异性^[25]^。例如，Alexey团队使用磷酸胍（phosphoryl guanidine, PG）及磷酰胺苯并唑寡核苷酸修饰的引物检测*KRAS*突变，其中PG修饰的检测限低至0.1%且特异性良好，可作为提升AS-PCR检测性能的通用手段^[31,32]^。

#### 2.1.3 微滴式数字PCR（droplet digital PCR, ddPCR）、芯片式数字PCR（chip digital PCR, cdPCR）与BEAMing（beads, emulsion, amplification, and magnetics）技术

数字PCR（digital PCR, dPCR）技术将DNA分配到众多微反应器中（每器模板≤1），依据扩增后阳性反应器的荧光信号比例精准定量原始溶液的核酸浓度^[33]^。与qPCR相比，其具有更高的灵敏度和特异性^[34]^。目前，dPCR主要应用于液体活检，成熟的商业平台有ddPCR和cdPCR两大类。此外，结合了磁珠捕获与流式技术的BEAMing技术因超低频突变的优异表现同样备受关注。

ddPCR通过乳化技术形成微小液滴作为反应器，因操作、仪器与方案最为简单，具有良好的通用性与推广性，是dPCR的主流技术^[35]^。Maeda团队和Pender团队^[36,37]^开发的ddPCR方法，分别实现了*KRAS*突变检测的1-10 ng的极低模板要求以及0.05%的检测限。一项前瞻性研究^[38]^表明，ddPCR检测NSCLC患者血浆中*KRAS*突变敏感性达64%，特异性和阳性预测值高达100%。同时，在诊疗领域，应用ddPCR检测早期患者血浆ctDNA的状态被证实可预测*KRAS*突变NSCLC患者免疫治疗和靶向治疗的疗效^[39,40]^。

cdPCR使用预制芯片的物理微孔阵列作为反应单元，相较ddPCR，其灵敏度更具优势，但较高的成本与操作难度导致其临床普及率较低^[41]^。BEAMing显著提升dPCR的检测性能，与ddPCR、AS-PCR和NGS比较，灵敏度最高，检测限达0.03%^[42,43]^。然而，其复杂的操作流程和极其昂贵的成本限制了其在临床场景中的进一步推广与应用。

单通道是dPCR方法不可忽视的局限性，但如今，许多多重dPCR技术的研发实现了高效同步检测KRAS在内的多种突变^[44,45]^。随着技术的不断创新与完善，dPCR在NSCLC精准诊疗中的应用前景将愈发广阔。

#### 2.1.4 高分辨率熔解曲线分析技术（high-resolution melting curve, HRM）

HRM根据野生型和突变型DNA熔解曲线（PCR体系荧光强度随温度的变化）形态的差异快速识别基因突变^[25]^。荟萃分析^[46]^证实了HRM检测KRAS基因突变的有效性，总体诊断敏感性、特异性、DOR、AUC分别为99%、96%、1121.36和0.996。有研究^[47,48]^证实了HRM与NGS检测结直肠肿瘤*KRAS*突变的一致性以及和焦磷酸测序灵敏度的相近性。尽管HRM无法通过识别DNA序列的变化确定具体突变类型（如G12C），阳性结果仍需进一步确认，但其简单高效、低成本和高灵敏的特点使其在肿瘤的快速筛查领域具有巨大的发展潜力^[47]^。同时，已有研究^[49-51]^通过结合HRM与dPCR来实现KRAS基因分型、KRAS和BRAF同时分型，并在液体活检的应用中取得不错结果。随着技术的更新与突破，HRM将为精准医学提供更高效、经济的技术支撑。

### 2.2 测序方法

#### 2.2.1 双脱氧测序法（Sanger测序）

Sanger测序基于链终止反应原理，利用荧光标记和毛细管电泳检测模板DNA序列^[25]^。该方法准确性高，能够检测未知突变，但与NGS和qPCR相比，其灵敏度较低，检测限约15%^[52]^。此外，通量低、成本高等特点也使其难以广泛普及。为提高检测性能，多种改进技术问世。例如，使用锁定核酸探针的野生型阻断循环测序方法（LNA-Sanger测序）可将低频突变的检测限提升至5%，野生型阻断实时聚合酶链反应（WTB-qPCR）联合Sanger测序能在单细胞水平检测组织和液体样本中的*KRAS*突变^[53,54]^。尽管NGS在灵敏度和特异性上已展现优势，但Sanger测序凭借技术成熟、操作相对简便等特点，在KRAS基因检测中仍占据重要地位，并在改进中不断提升。

#### 2.2.2 NGS

NGS是一种高通量技术，通过边合成边测序，单次即可完成数百万条DNA片段的序列测定，能全面检测KRAS基因突变、伴随突变和未知突变，相较Sanger测序所需模板DNA更少、速度更快、具备更高的通量和灵敏度^[55]^。有研究^[56]^利用能力验证样本对比NGS和非NGS技术在检测*KRAS*突变的性能，证实NGS方法总体可接受率高达98.8%。一项荟萃分析^[43]^纳入15项NGS检测配对组织和血浆KRAS样本的研究，合并敏感性、特异性、DOR、AUC分别为65%、88%、14.61、0.8574，尽管略低于dPCR的检测性能，总体上仍具有很高的准确性。另一项荟萃分析^[57]^表明，基于血浆样本的*KRAS*突变检测在NSCLC患者中总体具有很高的准确性，其中NGS最佳（敏感性、特异性、DOR、AUC分别为73%、64%、82.60、0.9162）。综上，NGS的*KRAS*突变检测能力在多项大样本研究中得到证实，已成为肿瘤临床分子诊断中不可或缺的重要技术。

#### 2.2.3 焦磷酸测序技术

焦磷酸测序技术基于DNA合成光反应原理，检测发光信号的有无及峰值来确定碱基种类及突变等位基因比例^[58]^。该技术在检测性能上表现突出：对于*KRAS*突变基因的检测限达到5%，明显优于Sanger测序，能检测出传统测序漏检的突变^[59-62]^；在与AS-PCR的对比中仍展现出高灵敏度，检测限达到1.25%-2.5%^[63]^。此外，该技术的应用场景还在拓展：与HRM技术的结合可进一步提高检测效率并降低成本^[48]^；而基于数字微流控的低成本便携焦磷酸测序平台，更能在30 min内快速检测丰度低至5%的*KRAS*突变^[64]^。未来，随着技术持续优化，焦磷酸测序有望在KRAS基因检测及肿瘤精准诊疗中发挥更大的作用。

### 2.3 基于成簇规律间隔短回文重复序列/CRISPR关联蛋白（clustered regularly interspaced short palindromic repeats/CRISPR-associated proteins, CRISPR/Cas）系统的诊断方法

CRISPR/Cas系统作为高效、低成本、可定制的基因编辑工具，在探索KRAS共突变基因致癌机制、耐药机制、表观遗传调控网络等研究^[65,66]^中已经得到广泛应用。通过完成目标DNA序列的切割，结合灵敏的检测方法，可以高特异性地检测*KRAS*突变。Cas12和Cas13已被证明可用于*KRAS*突变的检测，Zhou团队^[67]^开发了CRISPR-Cas12a检测*KRAS*突变的方法，检测限低至0.01%，且特异性良好，但临床意义仍需验证；Amintas等^[68]^开发了将AS-PCR与CRISPR-Cas13a相结合的CASPER方法，在检测灵敏度上达0.5%，优于AS-PCR，与dPCR相当，且具有定量能力，能检测出dPCR漏检样本。Shen等^[69]^开发了基于CRISPR-Cas9的选择性指数扩增方法检测晚期胰腺癌患者的*KRAS*突变，组织活检结果与NGS和AS-PCR一致，并能在液体活检中准确、及时地监测患者化疗疗效。但作为新型技术，基于CRISPR/Cas系统的诊断方法在NSCLC基因诊断等更多场景中的应用价值还需进一步探索。

### 2.4 小结

*KRAS*突变的分子诊断技术近年取得了显著进展，AS-PCR和NGS等已成为主流诊断技术。PCR实验平台操作简单、检测速度较快，但对未知突变的检测能力存在局限，而NGS凭借其高通量和检测未知突变的优势，已成为指南推荐的核心工具。ddPCR和BEAMing等技术显著提升了低频突变检测能力（检测限可达0.03%-0.05%），在疗效预测和动态监测中展现出重要价值。此外，虽临床转化尚待验证，CRISPR/Cas系统等新兴技术有望进一步突破灵敏度与特异性瓶颈。因此，结合检测需求与成本考虑，不同场景下优先选择的检测策略也会有所差异，例如初诊时为全面了解肿瘤基因状态，NGS或成本相对较低的多基因AS-PCR检测可成为优先选择；而动态监测及疗效评估时，具有液体活检优势的ddPCR将更为适合。诊断技术的精准化与多元化正极大推动*KRAS*^G12C^突变NSCLC个体化的诊疗进程（表1）。

**表1:** *KRAS*突变主要诊断技术比较

Technology	Limit of detection	Key advantages	Key limitations	Clinical application/status
ARMS-PCR	<1%	Rapid, sensitive, specific, low cost, mature system	Cannot detect unknown mutations; requires primer optimization	Mainstream technique for rapid detection of common mutations
ddPCR	0.05%	Extremely high sensitivity & specificity; absolute quantification	Traditionally single-plex (though multiplex is evolving)	Efficient liquid biopsy technology for efficacy prediction and monitoring
BEAMing	0.03%	Ultra-high sensitivity	Complex operation, extremely high cost	Research on ultra-low frequency mutations; limited clinical adoption
NGS	-	High-throughput, detects unknown and concomitant mutations	Performance in liquid biopsy slightly inferior to ddPCR	Core guideline-recommended tool, gold standard in clinical diagnosis
Pyrosequencing	1.25%-5%	Sensitivity superior to Sanger sequencing; quantitative analysis	-	Detects mutations missed by traditional sequencing; portable platforms enable rapid testing
Sanger sequencing	5%-15%	Detects unknown mutations; mature technology	Low sensitivity, low throughput	Important supplementary method, still used especially with improvements
CRISPR/Cas	0.01%-0.5%	High specificity, efficient, low cost	Clinical value requires validation	Emerging technology with significant application potential

KRAS: Kirsten rat sarcoma viral oncogene; PCR: polymerase chain reaction; ARMS-PCR: amplification refractory mutation system-PCR; ddPCR: droplet digital PCR; BEAMing: beads, emulsion, amplification, and magnetics; NGS: next-generation sequencing; CRISPR/Cas: clustered regularly interspaced short palindromic repeats/CRISPR-associated proteins.

## 3 靶向治疗进展

### 3.1 已获批靶向药物单药

#### 3.1.1 索托拉西布（Sotorasib, AMG510）

索托拉西布作为首个获批的*KRAS*^G12C^靶向共价抑制剂，其喹唑啉酮核心结合S-IIP，丙烯酰胺与第12位半胱氨酸形成共价键，异丙基苯基填充H95/Y96/Q99隐秘口袋，将突变KRAS蛋白不可逆锁定在GDP无活性态以阻断下游信号，且对非G12C突变细胞的活性差异超过7000倍，为良好的安全性奠定基础^[70]^。

II期临床研究^[12]^显示，索托拉西布单药治疗经治晚期NSCLC患者的ORR为37.1%，mPFS和mOS为6.8与12.5个月。该药据此获美国食品药品监督管理局（Food and Drug Administration, FDA）批准用于*KRAS*^G12C^突变NSCLC后线治疗^[71]^。III期试验（对比多西他赛）^[13]^中，索托拉西布mPFS更优（5.6 *vs* 4.5个月）且耐受性更佳，但mOS无显著改善（10.6 *vs* 11.3个月），可能是因为患者退出率失衡和后续治疗交叉导致OS获益的稀释，亦提示了肿瘤可能对于靶向药物的快速获得性耐药现象。在生物标志物的探索中，KEAP1突变、核转录因子红系2相关因子2（nuclear factor erythroid 2-related factor 2, NRF2）高激活、STK11突变、甲状腺转录因子-1（thyroid transcription factor-1, TTF1）低表达关联不良预后，共济失调毛细血管扩张突变（ataxia-telangiectasia mutated, ATM）野生型、治疗后ctDNA早期清除、炎症型肿瘤免疫亚型与有利预后相关^[72]^。汇总多项临床试验数据（n=549）的研究^[73]^显示：70.7%的患者发生治疗相关不良事件（treatment-related adverse events, TRAEs），其中≥3级占26.8%，常见TRAEs包括腹泻、恶心、肝酶升高。结果显示，其总体安全性可控、停药率低，腹泻和肝毒性经剂量调整及支持治疗可有效控制；先前免疫治疗虽影响肝毒性发生率，但不改变总体安全可控性。

索托拉西布已在多项真实世界研究中显示临床获益，包括法国^[74]^（n=458）、意大利^[75]^（n=196）、德国^[76]^（n=163）、美国（n=128^[77]^；n=105^[78]^）及中国^[79]^（n=49）队列，但其疗效与安全性存在一定差异。各研究ORR介于26%-51%，mPFS为3.5-6.5个月，mOS为8.2-12个月。这种差异可能与患者基线特征及共突变状态密切关联——如法国研究中因脑转移比例高（38%）、体能状态差，mPFS和mOS较低；而中国研究基线条件较好，ORR达51%，或提示东亚人群疗效优势。此外，上述多项研究^[74-79]^均证实KEAP1或STK11共突变与较差生存结局有关，与临床试验结果^[72]^一致。安全性方面，3级以上TRAEs发生率为5.2%-17.2%，以肝酶升高最常见。虽不同研究停药率差异较大（4.3%-25%），但剂量调整可在不影响疗效的前提下改善耐受性。此外，有研究^[80]^通过对比多西他赛单药或联合治疗患者，表明索托拉西布在生存期上有优势且未增加严重安全性问题。

综上，索托拉西布临床研究^[12,13]^数据可观，在广泛NSCLC人群中的总体疗效和可控安全性已获验证，未来应结合分子分型等实施个体化治疗，并注重毒性管理，尤其警惕与免疫治疗序贯时的肝毒性风险。

#### 3.1.2 阿达格拉西布（Adagrasib, MRTX849）

相较索托拉西布，阿达格拉西布在结构设计上扩大疏水基团、引入柔性侧链、增强S-IIP结合能力、降低P-糖蛋白外排并改善脂溶性，使其具有较强血脑屏障（brain blood barrier, BBB）通透力和更长的半衰期（23 *vs* 5 h）^[81,82]^。

在II期临床试验^[14]^中，经治*KRAS*^G12C^突变NSCLC患者ORR为42.9%，mPFS和mOS分别为6.5和12.6个月，33%脑转移患者的病灶缩小，与BBB穿透优势相符。常见TRAEs以胃肠道不良反应为主，≥3级TRAEs发生率为44.8%，多数经支持治疗或剂量调整缓解，据此获FDA批准用于该人群后线治疗^[83]^。III期初步结果^[84]^显示，其疗效（ORR为31.9%，mPFS为5.5个月）优于多西他赛（ORR为9.2%，mPFS为3.8个月），TRAEs发生率略高（94% *vs* 86.4%），但停药比例更低（7.7% *vs* 14.3%）。其安全性信号可能与其结构改进有关：优越的BBB渗透力和较长半衰期带来广谱组织分布，使依赖RAS信号通路更新修复的正常组织承受持续抑制压力，增加全身不良事件风险；同时作为细胞色素P450 3A4酶底物且清除缓慢，在合并用药时易蓄积，加剧毒性负担。

有间接比较研究^[85]^显示，其mPFS略优于索托拉西布，OS随时间趋于一致。生物标志物分析^[86]^提示，KEAP1突变（mOS：5.4 *vs* 19.0个月）、STK11突变（mOS：9.8个月 *vs* 未达到）和NRF2高表达（mOS：9.8 *vs* 19.0个月）与不良预后有关。这些结果与索托拉西布研究^[72]^相符合，提示相关分子的不利影响具有非特定药物的一致性，可能涉及抗氧化应激反应通路及肿瘤免疫微环境重塑，从而削弱KRAS抑制带来的信号阻断效果。

综上，阿达格拉西布通过结构优化实现了靶点结合力、BBB渗透力和药代稳定性的平衡，这些改进与其疗效尤其是颅内病灶控制密切相关，其安全谱可管理但需重视，共突变疗效差异提示精准分层的重要性。目前仍需多中心、大样本真实世界数据验证其长期获益与风险概况，并进一步阐明伴随基因状态对疗效的影响。

#### 3.1.3 氟泽雷塞（Fulzerasib, IBI-351/GFH925）

氟泽雷塞在索托拉西布基础上进行刚性骨架改造，以增强酸性环境的稳定性和药物活性，半衰期为17.4 h且达峰时间快，肝脏细胞色素酶代谢参与有限，有助于减少潜在药物相互作用并改善安全性^[87]^。II期研究^[15]^中，600 mg每日两次方案的后线ORR达49.1%，mPFS为9.7个月，TRAEs以贫血、肝酶升高和疲劳为主，≥3级发生率为41.4%且多数可管理。分子亚组分析显示STK11、KEAP1等共突变与显著缩短mPFS相关，这一模式与上述药物^[72,86]^结果一致。基于上述结果，国家药品监督管理局（National Medical Products Administration, NMPA）批准氟泽雷塞用于*KRAS*^G12C^突变NSCLC后线治疗，使之成为中国首款获批的*KRAS*^G12C^靶向药^[88]^。

刚性骨架不仅提高其稳定性，也有助于维持与KRAS蛋白的结合，从而保持起效快与药物浓度维持的平衡，加之每日两次方案的优化策略，使接近10个月的mPFS成为同类最佳^[12-17]^。同时，因较少依赖肝脏细胞色素酶清除^[87]^，其安全谱^[15]^呈现不同于阿达格拉西布^[83]^/索托拉西布^[73]^的一面：其胃肠道事件相对可控，而造血系统毒性比例较高，可能与分子脱靶或长期全身暴露有关，具体机制仍需进一步验证。此外，STK11和KEAP1共突变一贯预后不良，提示需要联合策略以改善这部分患者结局^[15,72,86]^。

#### 3.1.4 格索雷塞（Garsorasib, D-1553）

格索雷塞较低的亲脂性能减少非特异组织蓄积并提高血浆游离药物比例，有助于在控制P-糖蛋白外排敏感性同时增强BBB净通透率^[89,90]^。这一策略区别于阿达格拉西布的“强疏水、弱外排”路径^[81]^，尽管在脑转移小鼠模型中已取得与之相近的结果，仍需人体的专门评估以确认该潜力。II期研究^[16]^显示后线ORR达到50%，mPFS为7.6个月，TRAEs发生率为95%，≥3级占50%，主要为肝损害、贫血、胃肠道反应且多数可控，STK11、KEAP1、TP53等预示不良结局。

在同类靶向药物中，格索雷塞反应率处于较优水平，但疾病控制持续时间略短，尽管无患者因TRAEs停药^[16]^，长期用药监测仍需关注。STK11、KEAP1共突变延续了跨平台观察到的不良预后模式，而TP53变异的负性结果^[16]^提示可能通过基因组不稳定等途径加速耐药过程，有待进一步验证。基于II期结果，该药已获NMPA批准用于*KRAS*^G12C^突变NSCLC后线治疗，为今后开展未来精准分层及特殊人群方案提供依据。

#### 3.1.5 戈来雷塞（Glecirasib, JAB-21822）

戈来雷塞以腈基修饰的新骨架从源头控制整体亲脂性、建立新氢键网络，引入多卤素封闭易感代谢位点以提升稳定性和口服生物利用度，并将主要清除途径转向葡萄糖醛酸化^[91]^。IIb期研究^[17]^表明后线ORR为47.9%，mPFS为8.2个月，mOS为13.6个月，TRAEs发生率为97.5%，≥3级为38.7%，贫血、胆红素升高、肝酶升高常见，5%患者停药。分子分析提示KEAP1而非STK11突变与不良预后相关。基于此结果，该药已获NMPA批准用于*KRAS*^G12C^突变NSCLC后线治疗，为患者提供新的选择。

戈来雷塞的结构优化实现了高活性、优良药代动力学以及较低细胞色素酶依赖性的平衡，这支撑了接近50%的ORR和超过8个月的mPFS^[17]^。同时，其清除路径区别于同类，一方面降低了细胞色素酶代谢相关毒副作用以及药物相互作用风险，另一方面又使胆红素升高成为需要重点监测的不良反应信号。值得注意的是，STK11状态未显示明显疗效差异，不同于以往观察到的跨平台模式^[15,17,72,86]^，提示其对特定分子背景患者可能存在相对优势，但具体机制仍需前瞻验证（表2）。

**表2:** 已获批*KRAS*^G12C^直接靶向药物在NSCLC中的安全性及疗效

Author	Year	Drug	Study type	*n*	ORR (%)	mDOR (mon, 95%CI)	mPFS (mon, 95%CI)	mOS (mon, 95%CI)	TRAEs (%, ≥grade 3)	Common TRAEs (≥grade 3)	Discontinuation due to TRAEs (%)
de Langen AJ *et al*.^[13]^	2023	Sotorasib	III	171	28.1	8.6 (7.1-18.0)	5.6 (4.7-7.8)	10.6 (8.9-14.0)	33.0	Diarrhea 12%, ALT rise 8%, AST rise 5%	10.0
Skoulidis F *et al*. ^[12]^	2021	Sotorasib	II	126	37.1	11.1 (6.9-NR)	6.8 (5.1-8.2)	12.5 (10-NR)	20.6	ALT rise 6.3%, AST rise 5.6%, diarrhea 4%	7.1
Wislez M *et al*.^ [74]^	2025	Sotorasib	Retrospective study	458	33.2	3.8 (3.0-5.0)	3.5 (3.1-4.2)	8.3 (7.5-9.3)	-	γ-GT rise 4.0%, ALT rise 2.8%, AST rise 2.0%	16.5
Stratmann JA *et al*.^[76]^	2024	Sotorasib	Retrospective study	163	38.7	7.9 (4.9-10.8)	4.8 (3.9-5.9)	9.8 (6.5-NR)	17.2	Hepatotoxicity 5.1%, diarrhea 4.9%	4.3
Zhou KI *et al*.^[77]^	2024	Sotorasib	Retrospective study	128	34.0	4.0 (2.2-6.5)	6.0 (4.3-8.1)	12.0 (10.2-16.5)	-	Diarrhea 12.5%, hepatotoxicity 10.9%, pneumonia 6.3%	25.0
Bao XY *et al*.^[79]^	2024	Sotorasib	Retrospective study	49	51.0	-	6.5 (4.6-8.4)	10.7 (5.8-15.6)	7.6	Hepatotoxicity 3.8%, fever 3.8%	7.6
Passiglia F *et al*.^[75]^	2024	Sotorasib	Retrospective study	196	26.0	5.7 (4.4-7.0)	5.8 (5.0-6.5)	8.2 (6.3-9.9)	16.5	ALT/AST rise 12.2%, γ-GT rise 3%	4.6
Thummalapalli R *et al*.^[78]^	2023	Sotorasib	Retrospective study	105	28.0	-	5.3 (3.6-6.6)	12.6 (8.3-NR)	16.2	Hepatotoxicity, diarrhea	13.3
Mok TSK *et al*.^[84]^	2024	Adagrasib	III	301	31.9	8.3 (6.1-10.4)	5.5	-	47.0	-	7.7
Jänne PA *et al*.^[14]^	2022	Adagrasib	II	116	42.9	8.5 (6.2-13.8)	6.5 (4.7-8.4)	12.6 (9.2-19.2)	44.8	Anemia 14.7%, pneumonia 12.1%, hepatotoxicity 10.4%	6.9
Zhou Q *et al*.^[15]^	2024	Fulzerasib	II	116	49.1	-	9.7	-	41.4	Anemia 11.2%, hepatotoxicity 7.8%, γ-GT rise 7.8%	7.8
Li Z *et al*.^[16]^	2024	Garsorasib	II	123	50.0	12.8	7.6	-	50.0	AST rise 17%, ALT rise 15%, γ-GT rise 23%	0
Li Z *et al*.^[90]^	2023	Garsorasib	I	79	40.5	7.1	8.2	-	38.0	γ-GT rise 21.5%, ALT rise 10.1%, AST rise 8.9%	1.3
Shi Y *et al*.^[17]^	2025	Glecirasib	II	119	47.9	-	8.2	13.6	38.7	AST rise 10.9%, ALT rise 10.9%, hyperglyceridemia 7.6%	5.0

NSCLC: non-small cell lung cancer; *n*: number of patients with *KRAS*^G12C^ mutant NSCLC; ORR: objective response rate; mDOR: median duration of response; mPFS: median progression-free survival; mOS: median overall survival; 95%CI: 95% confidence interval; TRAEs: treatment-related adverse events; ALT: alanine aminotransferase; AST: aspartate aminotransferase; γ-GT: γ-glutamatranspeptidase; NR: not reported.

### 3.2 在研靶向药物单药

除已上市品种外，多款*KRAS*^G12C^抑制剂处于临床开发阶段，并在一线尝试、机制创新与耐药后再挑战等难题中有所突破。虽多数仍采用不可逆共价锁定S-IIP口袋这一经典策略，但在分子结构和作用方式上已出现明显分化，目前已有多个候选药物展现出令人鼓舞的临床潜力。

Divarasib（GDC-6036）通过优化S-IIP口袋匹配度和疏水相互作用，在体外活性和选择性上较索托拉西布/阿达格拉西布提升5-20倍和50倍^[92]^。I期结果^[93,94]^显示后线ORR达55.6%，mPFS达13.8个月，同时仅16.9%发生3级以上TRAEs，在疗效和安全性上有望达到同类最佳水平。高选择性的结构特点不仅可能延缓毒副作用发生，也为联合治疗提供了安全窗口。

Opnurasib（JDQ433）^[95,96]^和Olomorasib（LY3537982）^[97]^也均采用传统抑制模式，并显示出可靠的临床活性和可管理的安全性特征。特别值得注意的是，Olomarasib对既往接受过*KRAS*^G12C^抑制剂治疗的患者仍显示39%的ORR，为耐药患者提供了新的治疗选择^[97]^。HS-10370与GEC255均为高度选择性的共价小分子，开启了一线治疗的尝试，其中HS-10370在NSCLC中一线ORR达54.2%且mPFS超11个月，同时停药率极低^[98]^；而GEC255在早期试验^[99]^显示出高反应水平，不过其可重复性有待大样本验证。

D3S-001在机制层面^[100]^有所突破，其不仅针对静息GDP态KRAS蛋白，还能显著降低细胞内活化GTP-KRAS水平，双态高效耗竭细胞内KRAS蛋白。早期临床数据^[100]^显示，其在初治患者中ORR为66.7%，6个月PFS率达65.3%，更值得注意的是，该药物在已耐药患者中仍能实现40%的缓解率，表明其具有克服继发耐药的潜力。此外，MK-1084开发思路倾向于联合免疫应用，其改善代谢稳定性的同时保留良好组织渗透，使得与帕博利珠单抗联用时ORR可达74%，mPFS长达25个月，远高于单用水平，揭示了免疫联合策略在一线治疗中的广阔前景^[101]^（表3）。

**表3:** 其他*KRAS*^G12C^直接靶向药物在NSCLC中的安全性及疗效

Author	Year	Drug	Study type	*n*	ORR (%)	mDOR (mon, 95%CI)	mPFS (mon, 95%CI)	mOS (mon, 95%CI)	TRAEs (%, ≥grade 3)	Common TRAEs (≥grade 3)	Discontinuation due to TRAEs (%)
Sacher AG *et al*.^[93,94]^	2025	Divarasib	I	65	55.6	18.0 (11.1-24.9)	13.8 (9.8-25.4)	-	16.9	Diarrhea 3.1%, ALT rise 6.2%, AST rise 4.6%	4.6
Heist RS *et al*.^[97]^	2024	Olomorasib	I/II	58	40.0	-	9.0	-	5.0	Diarrhea, fatigue, nausea	0
Cassier PA *et al*.^[96]^	2023	JDQ443	I	38	41.7	-	-	-	7.1	Fatigue, edema, diarrhea, nausea	-
Cho BC *et al*.^[100]^	2025	D3S-001	I	25	66.7	-	-	-	16.7	-	0
Zhang Y *et al*.^[99]^	2023	GEC255	I	6	83.3	-	-	-	8.2	Diarrhea, ALT rise, rash, anemia	-
Sacher AG *et al*.^[101]^	2025	MK-1084	I	21	38.0	8.0	-	-	9.0	ALT rise	-
Meng XJ *et al*.^[98]^	2025	HS-10370	I	48	54.2	13.5	11.3	-	22.2	AST rise 3.2%, QT interval extension 3.2%	1.6

### 3.3 联合用药

#### 3.3.1 *KRAS*^G12C^抑制剂联合化疗

化疗能快速降低肿瘤负荷并减少早期耐药克隆，两者联用旨在兼顾短期缩瘤与延缓获得性耐药。II期研究^[22]^中，一线索托拉西布联合卡铂/培美曲塞疗法的ORR达88.9%，mPFS为6.6个月，mOS超过20个月，在程序性死亡配体1（programmed cell death ligand 1, PD-L1）表达<1%的人群获益尤为显著，主要≥3级TRAEs为血液学毒性。TP53突变、EGFR和MET扩增与获得性耐药有关。该疗法良好的疗效和安全性为PD-L1低表达或需快速缩瘤患者提供了重要途径，但高风险共突变人群可能需后续序贯策略巩固。

#### 3.3.2 *KRAS*^G12C^抑制剂联合ICIs

*KRAS*^G12C^抑制剂不仅直接阻断MAPK通路信号，还能通过上调主要组织相容性复合体I（major histocompatibility complex-I, MHC-I）抗原呈递分子、增强抗原加工与呈递相关基因表达、促进干扰素信号和趋化因子等多重机制改善肿瘤微环境，从而增强ICIs的疗效，这为*KRAS*^G12C^抑制剂与ICIs的协同提供了理论基础^[102]^。然而，多项研究^[103-105]^一致表明，免疫治疗与索托拉西布序贯治疗，尤其是间隔90天内会显著增加严重索托拉西布相关肝毒性和其他不良事件风险，因此用药时需格外警惕。

索托拉西布在联合ICIs的多队列Ib期研究中^[106]^，各线治疗总ORR约29%，mOS为15.7个月，同步组≥3级肝酶升高率显著高于序贯组，而后者安全性相对可控。阿达格拉西布与ICIs联合^[19]^的一线治疗ORR为44.3%，mPFS为11个月，其中高PD-L1（≥50%）亚组一线ORR达59%，mPFS接近28个月，但70%患者出现≥3级TRAEs，超40%患者^[107]^需停用任一方案。这提示该组合虽有深度缓解潜力，但安全管理挑战较大，尤其是在高表达人群以外的应用价值仍需权衡。Olomorasib联合ICIs的一线人群ORR为70%^[108]^，其中高PD-L1亚组的ORR为82%，肝损害为主要的不良反应。此外，前文提及的MK-1084与帕博利珠单抗的一线探索^[101]^在已观察到74%的早期反应率同时，重度不良事件比例低于阿达格拉西布/索托拉西布，为未来优化安全窗口提供可能。这些结果表明，高PD-L1表达患者从该组合中获益最大，而对于低至中表达或伴随复杂共突变的人群，应结合疾病控制需求、耐受风险及给药方法综合决策，以实现最佳平衡。

#### 3.3.3 *KRAS*^G12C^抑制剂联合SHP2抑制剂

SHP2是位于RAS上游的致癌型酪氨酸磷酸酶，是多种RTK信号向RAS-MAPK轴传递的关键中枢。*KRAS*^G12C^抑制剂治疗过程中，常见耐药机制之一是通过RTKs介导的旁路通路重新激活下游MAPK信号，从而削弱抑制效果。SHP2抑制剂可阻断这一旁路激活环节，并通过抑制GDP向GTP转换，提高KRAS-GDP占有率，使共价型抑制剂更易结合靶点，从源头延缓或逆转继发性耐药^[8,18]^。

临床上，多项不同分子组合验证了该策略在NSCLC中的可行性与差异化表现：索托拉西布联合RMC-4630^[109]^在经治人群中ORR为27%，主要严重TRAEs为外周/局部水肿和腹泻，可经调整控制；戈来雷塞和JAB-3312一线联合的ORR达到72.5%，但≥3级贫血及转氨酶升高较常见^[21]^；TNO155+JDQ433^[110]^在既往用过或未用过靶向*KRAS*^G12C^抑制剂人群后线治疗的ORR均为33%，但首次应用靶向药物患者的疾病控制率更高，中性粒细胞减少是主要高级别TRAEs；Divarasib+Migoprotafib的I期结果^[111]^显示，初治患者ORR为43.8%、mPFS超15个月，不良反应以腹泻和肝酶升高突出。这些研究提示，在伴随强RTK驱动或易发生通路再激活风险的人群中，引入SHP2抑制剂联合可能会带来额外获益，但结论仍需更大样本研究及机制分析进一步证实。

#### 3.3.4 *KRAS*^G12C^抑制剂联合EGFR单抗

EGFR通路改变是索托拉西布单药治疗的获得性耐药机制，其可绕过KRAS直接驱动下游信号通路，恢复肿瘤细胞增殖与存活^[18]^。抗EGFR单克隆抗体联合有望切断这一补偿性信号，通过双重封锁EGFR与KRAS同时抑制上游旁路和突变靶点本身，从而更全面地抑制肿瘤信号通路并延缓耐药发生^[12,97]^。索托拉西布+帕尼单抗在晚期NSCLC患者中后线治疗的ORR为45%、主要≥3级不良事件为肝酶升高且总体可控，仅少数患者需停用任一药物^[20]^。而氟泽雷塞+西妥昔单抗一线治疗^[112]^的ORR达到80%、mPFS为12.5个月，对脑转移亚组ORR高达71.4%，不良反应以皮疹、乏力、瘙痒常见，高等级TRAEs发生率约14.9%。这些结果表明，通过“双闸门”式阻断MAPK再激活链条，不仅有望提升初始缓解深度，还可能在良好可控的安全性前提下改善对远处转移尤其是脑转移灶的控制效果（表4）。

**表4:** *KRAS*^G12C^靶向药物与其他药物联合的安全性及疗效

Author	Year	*KRAS*^G12C^ inhibitor	Combined drug	Study type	Treatment line	*n*	ORR (%)	mPFS (mon)	mOS (mon)	TRAEs (%, ≥grade 3)	Any drugs discontinuation due to TRAEs (%)	KRAS^G12C ^inhibitors discontinuation due to TRAEs (%)
Zhao J *et al*.^ [21]^	2024	Glecirasib	JAB-3312 (SHP2i)	I/IIa	1	80	72.5	-	-	41.9	7.8	-
Negrao MV *et al*.^[110]^	2023	JDQ443	TNO-155 (SHP2i)	Ib/II	≥2	24	33.3	-	-	36.0	-	-
Falchook G *et al*.^[109]^	2022	Sotorasib	RMC-4630 (SHP2i)	I/II	≥2	11	27.0	-	-	29.0	14.3	4.8
Luo J *et al*.^[111]^	2025	Divarsib	Migoprotafib (SHP2i)	I	1	48	43.8	15.2	-	43.2	-	2.7
Dragnev KH *et al*.^ [108]^	2025	Olomorasib	Pembrolizumab (ICIs)	I/II	1	43	70.0	-	-	-	-	-
Jänne PA *et al*.^[19,107]^	2025	Adagrasib	Pembrolizumab (ICIs)	II	1	149	44.3	11.0	18.3	70.4	43.6	20.1
Li BT *et al*.^ [106]^	2022	Sotorasib	Pembrolizumab/Atezolizumab (ICIs)	Ib	all	58	29.0	17.9	15.7	58.6	37.9	-
Sacher AG *et al*.^ [101]^	2025	MK-1084	Pembrolizumab (ICIs)	I	1	34	74.0	25.0	-	33.0	20.0	-
Sacher AG *et al*.^ [101]^	2025	MK-1084	ICIs+chemotherapy	I	1	22	41.0	-	-	58.0	17.0	-
Majem M *et al*.^[112]^	2025	Fulzerasib	Cetuximab (EGFRi)	II	1	47	80.0	12.5	-	14.9	6.4	-
Langer CJ *et al*.^ [20]^	2024	Sotorasib	Panitumumab (EGFRi)	Ib	≥2	40	45.0	5.8	-	-	-	-
Akamatsu H *et al*.^ [22]^	2025	Sotorasib	Carboplatin-pemetrexed	II	-	30	88.9	6.6	20.6	-	-	-

SHP2i: Src homology region 2 domain-containing phosphatase 2 inhibitor; ICIs: immune checkpoint inhibitors; EGFRi: epidermal growth factor receptor inhibitor.

### 3.4 小结

目前已获批的*KRAS*^G12C^抑制剂通过共价结合S-IIP及各自的结构优化，形成了差异化的临床特性，也塑造了独特的安全性谱。索托拉西布奠定了高选择性的基础^[70]^；阿达格拉西布通过增强疏水性和抑制P-糖蛋白外排，显著提高BBB穿透力^[81,82]^；氟泽雷塞采用刚性骨架降低对细胞色素酶的代谢依赖，实现了目前最长的mPFS^[12-17]^；格索雷塞通过降低亲脂性、增加游离药物比例，有望成为脑转移患者的另一选择^[89,90]^；戈来雷塞引入腈基新骨架，将主要清除途径转向葡萄糖醛酸化^[91]^，减少了基于细胞色素酶代谢的相关毒性。然而，它们均面临由靶点机制决定的共同挑战，如肝酶升高、胃肠道反应和血液学毒性等。同时，多项研究^[15,17,72,86]^一致显示，STK11、KEAP1、NRF2等共突变是显著的负性预后因素，凸显单药治疗的局限。

在研药物则致力于拓展治疗维度：Divarasib在活性和选择性上显著优于早期药物^[92]^，且安全窗口理想^[93,94]^；D3S-001突破单一GDP态锁定模式，可同时耗竭活化型GTP-KRAS，在初治和耐药人群中均显示出卓越潜力^[100]^。此外，多个分子正针对一线治疗、免疫联合及耐药逆转等方向推进，进一步丰富了个体化治疗的选择。

联合治疗已成为克服耐药和提升疗效的关键路径。与化疗联用，患者ORR高达88.9%^[22]^，为需快速缩瘤的患者提供重要选择，但需密切关注毒性；与ICIs联用，虽在PD-L1高表达人群中有显著获益，但肝毒性等不良反应风险升高，安全性管理面临严峻挑战^[19,103-105,107]^。此外，与SHP2抑制剂^[21,109-111]^或EGFR单抗^[20,112]^联用，通过阻断旁路再激活和上游信号补偿^[18]^，显示出逆转耐药及提升缓解深度的潜力，对脑转移患者疗效显著^[112]^，但各类组合毒性谱不同，尚需进一步优化。

总之，近年来*KRAS*^G12C^抑制剂的研发取得了重大突破，实现了对这一“不可成药”靶点的精准干预。各药物凭借差异化设计，在颅内转移、代谢稳定性及毒性谱等方面形成互补，为临床提供了多样选择。然而，耐药问题与靶点相关毒性仍是当前单药治疗的核心局限。未来仍需深入研究，以全面提升肿瘤患者的生存获益与生活质量。

## 4 总结与展望

近年来，*KRAS*^G12C^突变NSCLC的诊断与靶向治疗取得显著突破。*KRAS*^G12C^突变的检测技术不断优化，基于PCR的新方法（如ddPCR、BEAMing）及NGS显著提升了检测效能，液体活检为无创动态监测创造条件。在治疗领域，已有多种靶向药物获批用于后线治疗，且各种联合策略正不断涌现，为提升疗效和克服耐药带来了新希望。

然而，当前研究仍存在若干局限。首先，绝大多数关键临床研究集中于经治晚期患者，致使一线治疗策略缺乏高级别循证医学证据支持。尽管部分药物及联合方案在一线探索中展现出良好前景，当前关于靶向药物用于一线治疗的疗效与安全性格局仍存在较大不确定性，亟需更大规模研究予以验证。其次，索托拉西布的真实世界研究结果存在地域与人群差异，且普遍低于临床试验数据，提示严格筛选的临床试验人群不能完全代表真实世界患者，多种因素可能影响药物的实际应用效果。目前其他*KRAS*^G12C^抑制剂真实世界证据仍非常有限，亟待开展国际多中心真实世界研究，以深入探索未知影响因素。此外，已获批药物虽各具特点，但完全缺乏头对头比较研究，导致临床实践中最佳治疗选择尚不明确，特别是在脑转移患者及靶向治疗进展后等关键场景，仍需更多研究提供指引。同时，STK11、KEAP1等被一致认定为负性预后因素的共突变尚无针对性前瞻干预研究，无法基于分子特征实现个体化治疗策略选择。

展望未来，为进一步提升*KRAS*^G12C^突变NSCLC的诊疗水平，应致力于推动一线治疗及个体化联合策略的高级别临床研究，积极开展真实世界与头对头比较研究，并深入探索基于生物标志物的精准治疗路径。例如，可针对STK11/KEAP1共突变人群，前瞻性地评估特定联合方案对比标准治疗的疗效与安全性。同时，加强耐药机制研究、新型药物研发及联合治疗的安全性管理，也将为克服治疗瓶颈提供关键支持。随着基础研究与临床实践的深度融合，*KRAS*^G12C^突变NSCLC的治疗有望迈向更高疗效、更低毒性的时代。
